# Functional and self-rated health mediate the association between physical indicators of diabetes and depressive symptoms

**DOI:** 10.1186/1471-2296-15-157

**Published:** 2014-09-20

**Authors:** Sylvia Boehme, Christian Geiser, Babette Renneberg

**Affiliations:** Klinische Psychologie und Psychotherapie, Freie Universität Berlin, Habelschwerdter Allee 45, 14195 Berlin, Germany; Department of Psychology, Utah State University, 2810 Old Main Hill, Logan, UT 84322-2810 USA

**Keywords:** Chronic diseases, Depression, Diabetes, Health care, Self-rated health

## Abstract

**Background:**

Depression is common among persons with diabetes and associated with adverse health outcomes. To date, little is known about the causal mechanisms that lead to depression in diabetes. The aim of the present study was to examine to which extent functional and self-rated health mediate the association between physical health and depressive symptoms in diabetes.

**Methods:**

Data of *n* = 3222 individuals with type 2 diabetes were analyzed cross-sectionally and longitudinally at three measurement occasions using path analysis. Indicators of physical health were glycemic control, number of comorbid somatic diseases, BMI, and insulin dependence. Furthermore, functional health, self-rated health and depressive symptoms were assessed.

**Results:**

The effects of physical health on depressive symptoms were largely mediated by functional health and self-rated health. There was only a weak indirect effect of physical health on depressive symptoms. In contrast, self-rated health was a strong direct predictor of depressive symptoms. Self-rated health in turn depended strongly on patients’ functional health.

**Conclusions:**

The way individuals perceive their health appears to have a stronger effect on their depressive symptoms than objective physical indicators of diabetes. Therefore practitioners should be trained to pay more attention to their patients’ subjective health perceptions.

**Electronic supplementary material:**

The online version of this article (doi:10.1186/1471-2296-15-157) contains supplementary material, which is available to authorized users.

## Background

Depressive symptoms are highly prevalent in patients with diabetes [1]. Anderson et al. [1] reported a prevalence rate of depression twice as high in patients with diabetes than in a comparison group without diabetes. Patients with comorbid depressive symptoms have higher mortality rates [2], more diabetes-related complications [3], a decreased quality of life, [4] as well as a higher symptom burden [5]. Furthermore, depression especially in physical illness is challenging as people’s beliefs about depression may compromise depression screening and therapy [6]. Therefore, appropriately identifying and treating depressive symptoms in patients with diabetes is a very important, yet often neglected issue.

Although the high prevalence of depressive symptoms in patients with diabetes is well established, empirical evidence on a potential relationship between physical indicators of diabetes and psychological variables is inconsistent. While some studies found significant associations between depression and glycemic control e.g. [7, 8] others found no such relationship [9]. In a recent review, effects of antidepressive therapy on glycemic control in patients with diabetes and depression were contradictory [10]. Furthermore, the causal direction of the relationship between physical symptoms of diabetes and depressive symptoms remains unclear. A large meta-analysis found that depression may constitute a risk factor for diabetes [11] but there is also strong meta-analytic evidence for depression being a consequence of diabetes [12].

**Figure 1:**
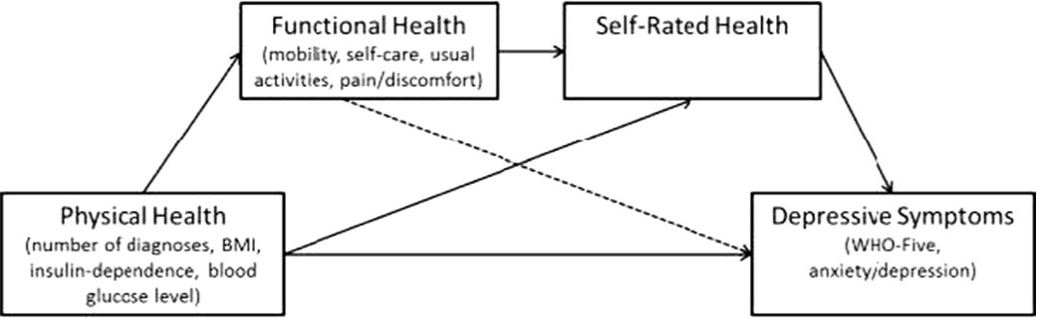
**Extended path model based on Whitelaw and Liang’s theoretical model with depressive symptoms as final mental health outcome of PH, FH, and SRH in type 2 diabetes.**

The goal of the current study was to learn more about the mechanisms underlying the relationship between depression and diabetes. Specifically, this study examines the relationship between physical indicators of diabetes, functional health (FH), self-rated health (SRH), and their effects on depressive symptoms In the present study, we adapted the theoretical model proposed by Whitelaw and Liang [13], in which PH, FH, and SRH are causally linked and FH serves as a mediator between chronic illness and SRH (see Figure 1). Compared to SRH, FH reflects a specific aspect of health-related quality of life (HRQoL). FH is defined as the ability of an individual to perform and adapt to his or her environment. We extended Whitelaw and Liang’s model by including mental health (i.e., depression) as the final outcome variable of PH, FH, and SRH. In addition to including depressive symptoms as a mental health outcome, we examined multiple disease-specific measures of PH as well as different indicators of FH. In the present article, we present analyses of cross-sectional as well as longitudinal data.

Strong associations have been reported for the relationship between SRH and depression among people with diabetes. SRH refers to an individual’s perception of his or her current health and has been shown to be a strong predictor of morbidity, hospitalization, and mortality in the elderly [14, 15]. In addition, a recent review found strong negative associations between health-related quality of life HRQoL; [16] and depression in people with diabetes [17].

Ali et al. [17] proposed that SRH should be examined as a mediator between diabetes and depressive symptoms in order to improve understanding of the mechanisms that lead to depression in diabetes. To the best of our knowledge, there has only been one study so far that has addressed this issue. Jang et al. [18] confirmed that SRH mediates the relationship between diabetes and depressive symptoms. In this study, however, the level of diabetes severity was not assessed.

Our study aimed at investigating the mechanisms of depression in diabetes within a framework of physical and functional health predicting SRH and depressive symptoms. We expected to see a higher prevalence of depressive symptoms in persons with diabetes compared to the general population. Furthermore, using the path model in Figure 1, we tested whether PH was directly associated with depressive symptoms above and beyond FH and SRH. Moreover, we tested the hypothesis that FH and SRH serve as mediators of the relationship between PH and depressive symptoms in diabetes.

## Methods

### Participants and Procedure

**Figure 2:**
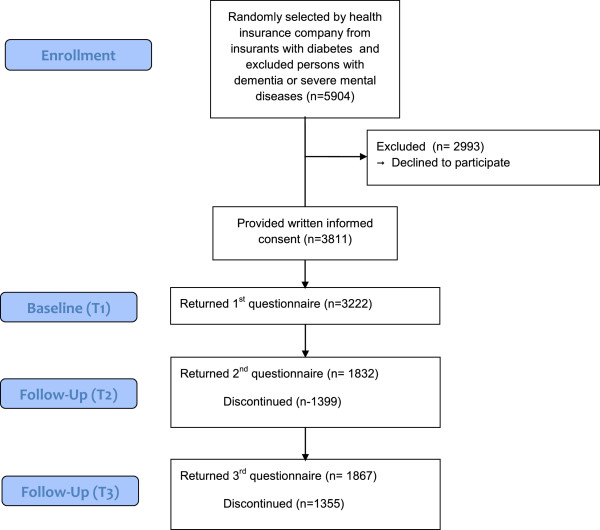
**Participants’ flow diagram according to CONSORT statement.**

Data from randomly selected insurants of a German health insurance company (Techniker Krankenkasse) meeting criteria for type 2 diabetes (according to their physician’s diagnosis) were used for the present analyses. The study was conducted according to the ethical guidelines of the insurance company. Authors obtained permission from the health insurance to analyze the de-identified data. Further ethics approval was not required as per German ethical guidelines. The questionnaires were sent by mail to the participants by the insurance company. Individuals who did not suffer from dementia or severe mental diseases, provided written informed consent, and returned at least the first of three questionnaires were included in the study. Measurements were taken at recruitment as well as four and ten months after recruitment, respectively. The following analyses are based on a subsample of 3,222 participants (male n = 2541, female n = 681, mean age = 68.11, *SD* = 7.70). Participants and non-participants (individuals who did not return the first questionnaire) did not differ in gender or number of diagnoses (*p* < .001) but participants were significantly younger than non-participants (67.08 vs. 69.70 years; *p* < 0.05). Comprehensive sample characteristics are presented in Table 1. A participants’ flow diagram is presented in Figure 2.

**Table 1:** **Sample characteristics**

	T1		T2		T3	
	valid *n*	*n (%)*	valid *n*	*n (%)*	valid *n*	*n (%)*
N	3222		1823		1867	
Gender	3222		1823		1867	
Female		681 (21.1)		380 (20.8)		382 (20.5)
Male		2541 (78.9)		1443 (79.2)		1485 (79.5)
Insulin Dependent	3222	1467 (45.0)	1823	869 (47.7%)	1867	924 (49.5)
Critical Blood						
Glucose Level	2458	1065 (32.6)	258	155 (60.1)	239	126 (52.7)
(HbA_1c_ >7.0%)						
Depressive Symptoms WHO-5^a^	2876		1633		1665	
<28 (indicates MDD)		546 (16.9)		269 (16.5)		320 (19.2)
<52 (poor emotional well-being)		591 (18.3)		327 (20.0)		314 (18.9)
		*M (SD)*		*M (SD)*		*M (SD)*
Age (years)	3140	68.11 (7.70)	1785	68.55 (7.48)	1826	68.47 (7.5)
Gender (women = 0, men = 1)	3222	.79 (.41)	1823	.79 (.41)	1867	.80 (.40)
BMI	3088	30.05 (5.38)	1768	30.18 (5.75)	1812	30.1 (5.5)
Blood Glucose Level in% (NGSP)	2458	6.92 (.89)	258	6.92 (.95)	239	6.87 (.84)
No. of diagnoses	3222	4.63 (1.53)		/		/
SRH^b^	2924	5.3 (1.95)	1627	5.49 (2.0)	1661	5.46 (1.96)
FH (Total Score)^c^	3160	.76 (.25)	1791	.76 (.25)	1528	.76 (.25)
Mobility^d^	3202	1.51 (.51)	1814	1.51 (.51)	1549	1.52 (.51)
Self care^d^	3200	1.13 (.37)	1811	1.13 (.37)	1547	1.14 (.38)
Usual activities^d^	3188	1.43 (.55)	1807	1.43 (.56)	1543	1.43 (.56)
Pain/discomfort^d^	3196	1.94 (.59)	1812	1.93 (.59)	1546	1.93 (.60)
Anxiety/depression^d^	3195	1.38 (.55)	1813	1.37 (.54)	1544	1.34 (.52)
Depressive Symptoms WHO-5 (total score)^a^	2876	56.72 (24.46)	1633	58.34 (24.30)	1665	57.01 (24.91)

^a^Range: 1–100; higher scores indicating better emotional well-being.

^b^Range: 1–10; higher scores indicating better SRH.

^c^Range: -0.207-1; higher scores indicate less functional impairment.

^d^FH Subscales Range: 1–3; higher scores indicate higher functional impairment.

A subset of 2,458 persons out of a total of 3,222 participants reported their blood glucose level (HbA_1c_) at first assessment. To test whether participants with and without available HbA_1c_ differed with regard to their mean scores on the relevant measures, we conducted detailed sensitivity analyses. We found significant differences in means (0.19 ≤ *d* ≤ 0.34) between those who reported their HbA_1c_ and those who did not^a^. To examine whether these differences would affect the results of the path analysis, we ran the analysis separately for the reduced sample of 2,458 persons (using listwise deletion of missing values) and the full sample of 3,222 persons (using full information maximum likelihood [FIML] estimation with missing data). Differences in results between the reduced and the full sample were marginal. Thus, we will report the results for the full sample with FIML estimation below.

### Measures

#### Physical health measures (PH)

As a measure of glycemic control, participants reported their blood glucose level (HbA_1c_) according to their latest laboratory test results. An HbA_1c_ > 7.0% was regarded as critical. HbA_1c_ was used as a continuous variable in the analyses. The number of comorbidities was calculated from insurance data by summing up all diagnoses from a list of 11 diseases that a participant had been diagnosed with in the previous 12 months before participation (arthrosis, cancer, hypertension, coronary heart disease, arteriosclerosis, myocardial infarction, chronic heart failure, stroke, COPD, asthma, diabetes). Furthermore, participants reported their insulin-dependence, weight and height.

### Self-Rated Health (SRH)

SRH was assessed using a well-established and validated single item measure e.g., [19]. Participants were asked to estimate their SRH on a scale ranging from 0 (“very poor”) to 10 (“very good”). The exact wording was: “If you were to rate your general state of health on a scale from 0 to 10, (“0” meaning “couldn’t be worse” and “10” meaning “couldn’t be better”), how would you rate your current state of health?”

### Functional Health (FH)

To assess FH, the following subscales of the EQ-5D were used: mobility, self-care, usual activities, and pain/discomfort (3-point scale from 1 = ‘no problems’ to 3 = ‘severe problems’). Subscale scores were computed according to the EQ-5D value sets [20]. Larger scores indicate fewer functional limitations. The subscales mobility, self-care, usual activities, and pain/discomfort were entered separately into our model. The subscale anxiety/depression was used as an indicator for the mental health outcome as described below.

### Depressive Symptoms

To assess depressive symptoms, we used the World-Health-Organization-Five scale (WHO-5), which is a brief and widely used measure of emotional well-being e.g., [21]. The WHO-5 has shown excellent internal consistency (Cronbach’s α = .91) and good external validity against SCID of 80% [22, 23]. Also, the comparative validity against physicians’ diagnoses showed to be excellent. While physician sensitivity for detecting major depressive disorder was only 40%, WHO-5 Screening identified 94% of patients with major depressive disorder [22]. According to the WHO a score < 52 indicates poor emotional well-being, and a score < 28 is regarded as an indicator of a major depressive disorder [22, 23]. Higher scores indicate fewer depressive symptoms. As a second indicator of depressive symptoms, we used the anxiety/depression subscale of the EQ-5D. According to the proposed model, depressive symptoms are regarded as the distal outcome. Therefore, the anxiety/depression subscale of the EQ-5D was used as an outcome variable instead of a predictor variable in terms of functional health.

### Statistical Analyses

The cross-sectional associations of PH, SRH, and FH with depressive symptoms in diabetes were examined by estimating the path coefficients of the proposed path analytic model using time 1 data only (see Figure 1). In the path model, the HbA_1c_ value, insulin-dependence, the number of diagnoses, and the BMI were used as separate indicators of PH. The EQ-5D subscales (except anxiety/depression) were used as separate indicators of FH. SRH was represented in the model by the single item measure. Finally, we included both the total WHO-5 score and the anxiety/depression subscale of the EQ-5D as separate indicators of the final depression outcome. Age and gender were included as covariates in the model. Dummy variables were created for gender (0 = women, 1 = men) and insulin-dependence (0 = not insulin-dependent, 1 = insulin-dependent). Our model was a “full forward model” [24], in which all direct paths from each construct to all other constructs following them in the proposed causal order shown in Figure 1 were estimated. The model also included all possible correlations between the exogenous physical symptoms variables and covariates as well as correlated residual variables for all endogenous variables at the same level in the model and thus represented a saturated model. To confirm the results found in the cross-sectional approach we ran additional longitudinal path analyses using a cross-lagged panel design [25] in which each variable was regressed on both its own previous measure(s) and the lagged measures of the other variables at previous measurement occasions. Longitudinal cross-lagged analyses allow for stronger tests of the proposed causal ordering of the variables. In our path analyses, we used FIML estimation to take all available data point into account [26]. In the cross-lagged panel model, the T1-measures of PH, the FH-subscales, SRH and the two measures of depressive symptoms as well as the covariates age and gender were included as separate indicators of following T2 and T3 measures: BMI, insulin dependence, the FH subscales, SRH, WHO-5 score and the anxiety/depression subscale of the EQ-5D. Due to the great amount of missing data for HbA_1c_ at later assessments the T2 and T3 measures of HbA_1c_ could not be included in the longitudinal analyses.

### Ethical approval

The data collection was conducted and ethically approved by the German health insurance company Techniker Krankenkasse. The current participants did not participate in any intervention over the course of the data collection.

## Results

A total of *N* = 3222 participants with diabetes were included in the study. Sample characteristics with means and standard deviations for all variables and assessment points are displayd in Table 1.

### Depressive Symptoms

Participants with type 2 diabetes showed a mean WHO-5-Score of 56.72 (*SD* = 24.46). According to WHO-5 definitions [22, 23], 16.9% of the sample showed severe depressive symptoms (WHO-5 scores < 28) and an additional 18.3% of the participants showed poor emotional well-being (WHO-5 scores < 52).

The zero-order correlations between the SH, FH, and PH measures and the two depression outcome variables at the first measurement occasion are presented in Table 2. Both measures of depression were substantially and significantly related to all measures of SH and FH. There were fewer and weaker correlations among the depression measures and measures of PH. The largest absolute correlation between PH and depression was found between the number of diagnoses and the WHO depression score (*r* = -.272, *p* < .001). The HbA_1c_ score was only marginally correlated with the WHO depression score (*r* = .088, *p* < .001) and not at all correlated with the EQ-5D anxiety/depression subscale (*r* = .025, n.s.). Slightly higher and statistically significant correlations were found between the BMI and these measures.

**Table 2:** **Pearson correlations of health constructs and physical indicators of diabetes with measures of depressive symptoms at first measurement occasion**

	Depressive symptoms (WHO-5) ^a^	Anxiety/Depression (EQ-5D-subscale)
Depressive Symptoms (WHO-5)^a^		-.54**
SRH	.58**	-.36**
Mobility	-.37**	.21**
Self-Care	-.33**	.24**
Usual Activities	-.52**	.35**
Pain/Discomfort	-.43**	.31**
BMI	-.17**	.10**
Blood Glucose Level	-.09**	.03
in mmol/mol (IFCC)		
in% (NGSP)		
No. of diagnoses	-.27**	.17**
Age	.12**	-.17**
Gender (women =0; men = 1)	.11**	-.13**

*Note.* ^a^Higher scores indicate a smaller level of depressive symptoms; **p < .01.

### Cross-sectional path analyses

**Figure 3:**
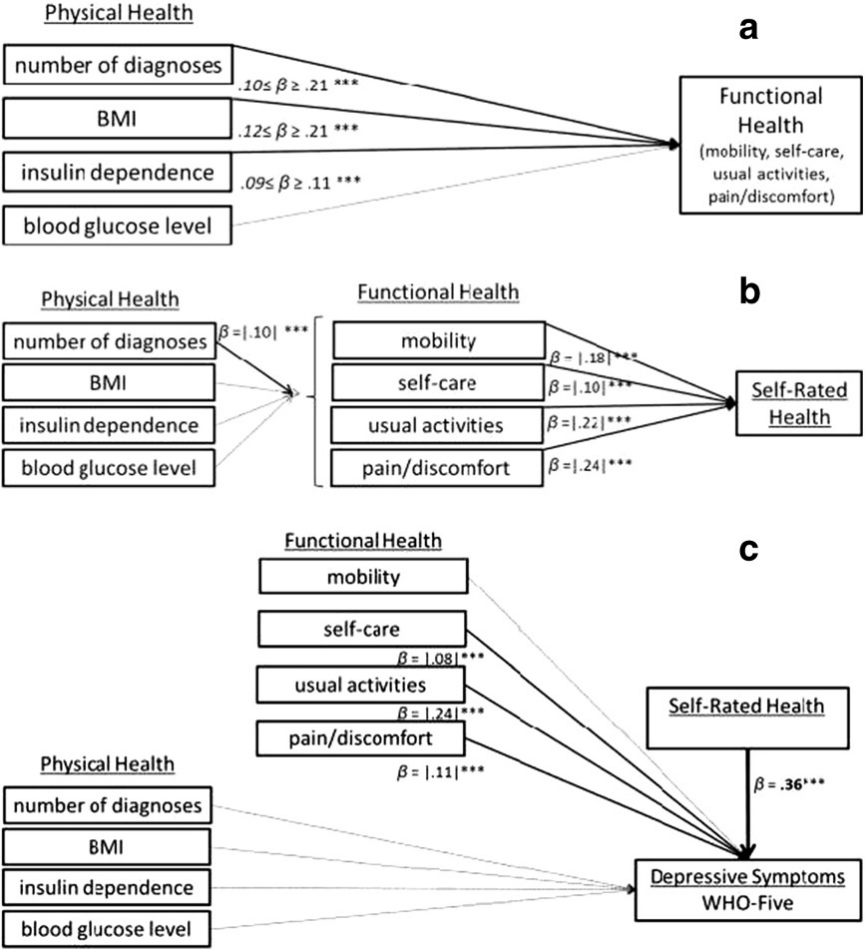
**Path models. a**. Path model Part I: FH outcomes. **b**. Path model Part II: SRH outcome. **c**. Path model Part III: Depressive Symptoms in terms of WHO-Five. *Note:* non- significant paths are displayed in a dotted line; ***p < .001.

Table 3 displays the results of the path analysis in terms of the estimated path coefficients and *R*^2^ values for each endogenous variable in the model. As displayed in Figures 3a and 3b, the number of diagnoses showed strong standardized path coefficients for predicting each of the four FH outcomes (*β* > 0.10). In addition, the BMI and insulin-dependence variables had significant effects on all four indicators of FH, whereas the blood glucose level (in terms of the HbA_1c_ value) did not have a significant effect on any of the FH outcomes. In total, between 4 and 11% of the variability in FH measures were explained by PH indicators in the model.

**Table 3:** **Estimated path coefficients, standard errors, significance tests, and measures of fit for the proposed path model**

Paths	*B*	*SE*( *B*)	β	*p*	*R* ^2^	*SEE*
Mobility on					.11	0.48
Number of diagnoses	0.07	0.01	0.20	< .001		
BMI	0.02	0.01	0.21	< .001		
Insulin-dependence	0.11	0.02	0.11	< .001		
Blood glucose level	-0.01	0.01	-0.01	.68		
Gender	-0.07	0.02	-0.06	< .001		
Age	0.00	0.00	0.06	.001		
Self-care on					.04	0.36
Number of diagnoses	0.02	0.02	0.10	< .001		
BMI	0.01	0.01	0.12	< .001		
Insulin-dependence	0.07	0.01	0.09	< .001		
Blood glucose level	-0.02	0.01	-0.04	.09		
Gender	-0.01	0.02	-0.01	.54		
Age	0.00	0.00	0.07	< .001		
Usual activities on					.09	0.53
Number of diagnoses	0.08	0.01	0.21	< .001		
BMI	0.02	0.00	0.15	< .001		
Insulin-dependence	0.11	0.02	0.10	< .001		
Blood glucose level	0.00	0.01	0.01	.82		
Gender	-0.13	0.02	-0.10	< .001		
Age	-0.00	0.00	-0.03	.07		
Pain/discomfort on					.09	0.57
Number of diagnoses	0.07	0.01	0.19	< .001		
BMI	0.02	0.00	0.17	< .001		
Insulin-dependence	0.12	0.02	0.10	< .001		
Blood glucose level	-0.01	0.01	-0.01	.56		
Gender	-0.18	0.03	-0.12	< .001		
Age	-0.00	0.00	-0.05	.01		
SRH on					.39	1.53
Mobility	-0.69	0.07	-0.18	< .001		
Self-care	-0.53	0.09	-0.10	< .001		
Usual activities	-0.78	0.07	-0.22	< .001		
Pain/discomfort	-0.80	0.06	-0.24	< .001		
Number of diagnoses	-0.12	0.02	-0.10	< .001		
BMI	-0.01	0.01	-0.03	.07		
Insulin-dependence	-0.14	0.06	-0.04	.02		
Blood glucose level	-0.10	0.04	-0.05	.007		
Gender	-0.07	0.07	-0.01	.34		
Age	0.03	0.00	0.10	< .001		
Depressive Symptoms (WHO-5)(WHO-5) on					.43	18.51
SRH	4.53	0.24	0.36	< .001		
Mobility	-0.64	0.87	-0.01	.46		
Self-care	-5.04	1.10	-0.08	< .001		
Usual activities	-10.48	0.84	-0.24	< .001		
Pain/discomfort	-4.67	0.72	-0.11	< .001		
Number of diagnoses	-0.33	0.25	-0.02	.18		
BMI	0.05	0.07	0.01	.44		
Insulin-dependence	1.17	0.75	0.02	.12		
Blood glucose level	-0.74	0.48	-0.03	.12		
Gender	2.64	0.87	0.04	.002		
Age	0.35	0.05	0.11	< .001		
Anxiety/depression on					.22	0.49
SRH	-0.06	0.01	-0.20	< .001		
Mobility	-0.05	0.02	-0.04	.04		
Self-care	0.12	0.03	0.08	< .001		
Usual activities	0.17	0.02	0.18	< .001		
Pain/discomfort	0.12	0.02	0.13	< .001		
Number of diagnoses	0.00	0.01	0.00	.97		
BMI	-0.00	0.00	-0.02	.36		
Insulin-dependence	-0.06	0.02	-0.06	.001		
Blood glucose level	-0.00	0.01	-0.00	.84		
Gender	-0.13	0.02	-0.09	< .001		
Age	-0.01	0.00	-0.16	< .001		

*Note. N* = 3222. *B* = unstandardized path coefficient. *SE* = standard error; β = standardized path coefficient; *SEE* = standard error of estimate (estimated standard deviation of the residual variable).

Furthermore, Figure 3b shows that indicators of FH were the strongest predictors of SRH with all four standardized path coefficients related to indicators of FH (*β* values between |0.10| and |0.24|). There were no gender differences in SRH once the other variables were controlled for in the analysis. Overall, the model accounted for 39% of the variability in SRH.

For the two final outcome measures of depressive symptoms, SRH was the strongest predictor of depressive symptoms (*β* > |0.20|; *p* < .001). Furthermore, the usual activities subscale used as an indicator of FH showed a relatively strong direct incremental effect on both indicators of depressive symptoms (*β* > |0.18|; *p* < .001). Figure 3c shows the results on depressive symptoms in terms of the WHO-Five measure.

In terms of the measures of PH, the path coefficients for number of comorbid diagnoses, BMI, and the HbA_1c_ score were consistently non-significant for both outcome measures supporting the mediating effect of FH and SRH in the association of PH and depressive symptoms. Insulin dependence had significant direct effects on the anxiety/depression subscale (*β* > |0.06|; *p* < .001) but not on depressive symptoms assessed via the WHO-Five. Overall, 43% of the variability in the WHO-5 scores and 22% of the variability in the anxiety/depression subscale scores of the EQ-5D were explained in the model. A separate analysis in which only the measures of PH were used as predictors of the depression scores revealed that the PH measures alone accounted for only 5.2% of the variability in the WHO-5 scores and only 1.3% of the variability in the EQ-5D anxiety/depression scores.

### Indirect effects

Table 4 shows the 95% confidence intervals for the indirect effects which were estimated based on bias-corrected bootstrapping [27]. The indicators of PH and FH were significantly indirectly related to SRH and depressive symptoms. Only blood glucose level did not show any significant indirect relation to either one of the outcome measures.

**Table 4:** **Indirect effects according to proposed paths in Figure** 1

	SRH			Depressive symptoms (WHO Score)			Depressive symptoms (anxiety/depression)		
	*Estimate*	95% CI	*SE*	*Estimate*	95% CI	*SE*	*Estimate*	95% CI	*SE*
PH indicators									
Number of diagnoses	-0.14*	(-.16; -.12)	.01	-0.17*	(-.19; -.15)	.01	.11	(.09; .13)	.01
BMI	-0.12*	(-.14; -.10)	.01	-0.12*	(-.15; -.09)	.01	.08	(.06; .10)	.01
Insulin-dependence	-0.08*	(-.10;-.05)	.01	-0.08*	(-.11; -.06)	.01	.06	(.04; .07)	.01
Blood glucose level	0.01^ns^	(-.02; .03)	.01	-0.01 ^ns^	(-.04; .02)	.01	.01	(-.02; .029	.01
FH indicators									
Mobility				-0.07*	(-.08; -.05)	.01	.04	(.03; .05)	.01
Self-care				-0.04*	(-.05; -.02)	.01	.02	(.01; .03)	.00
Usual activities				-0.08*	(-.10; -.06)	.01	.05	(.03; .06)	.01
Pain/ discomfort				-0.09*	(-.10; -.07)	.01	.05	(.04; 06)	.01

Note. Standardized parameter point estimates and bias-corrected bootstrapped (10.000 samples) CIs and SEs for indirect effects; CI = Confidence Interval: lower and upper boundaries; SE = Standard Error; *p < .001; ^ns^not significant.

### Longitudinal path analyses

To reduce complexity we only report significant and direct associations (beyond the autoregressions). For further details on the longitudinal results see Additional file 1: Table S1. The PH indicators number of comorbid diagnoses, insulin-dependence, and BMI predicted FH at T2. Blood glucose level did not predict FH longitudinally. At T3 of all previous PH variables only the number of diagnoses predicted two FH subscales (mobility and usual activities), and insulin-dependence predicted mobility. At T2 SRH was predicted by the number of diagnoses and insulin-dependence as well as by the FH subscales mobility and pain/discomfort. At T3 of all PH and FH indicators only the number of diagnoses predicted SRH. Depressive symptoms (WHO-5) at T2 were predicted by the number of comorbid diagnoses and the FH subscale self-care. At T3 the number of diagnoses and previous mobility predicted depressive symptoms (WHO-5). Of all PH, FH, and SRH indicators only the number of diagnoses predicted the anxiety/depression scale at T3 but not at T2, where no significant direct predictors for anxiety/depression were found beyond autoregressive effects. Furthermore, SRH was found to be the strongest predictor of depressive symptoms at T2 (for WHO-5 score and the anxiety/depression subscale).

## Discussion

The aim of our study was to shed more light on the causes of depression in diabetes. For this purpose, we considered both patients’ objective PH status and more subjective health constructs such as FH and SRH and studied their interplay within a large population using a complex path model adapted from Whitelaw and Liang [13].

### Summary of findings

As expected, the participants in our study showed a significantly higher level of depressive symptoms compared to the general population. With a mean score of 57 on a scale from 0–100 our study’s participants showed considerably more depressive symptoms than the general population for which Bech et al. [21] reports a mean score of 69. This is even more remarkable as our sample consists of a considerable high percentage of male participants who usually report less depressive symptoms than women. First, our results confirm Whitelaw and Liang’s proposed model [13] regarding the strong relationship between PH, FH, and SRH in diabetes. Second, our extension of their model to predicting mental health as a distal outcome helped us clarify that SRH and FH predict depressive symptoms in diabetes and that these constructs to a large extent mediate the effect of PH on depressive symptoms in diabetes. Altogether, our cross-sectional model accounted for over 40% of individual differences in depressive symptoms as measured by the WHO-5 scale, which can be seen as a large effect. In contrast, PH measures alone accounted for only between 3.5 and 9.5% of the variability in depression. Moreover, we were able to confirm Whitelaw and Liang’s model for diabetes with more differentiated indicators of PH than those used by Whitelaw and Liang and confirmed the cross-sectional results also longitudinally. The longitudinal analyses also showed previous SRH to be the strongest predictor of depressive symptoms at second assessment (T2) whereas PH had an at most modest longitudinal association.

Study results concerning the causal mechanisms underlying the relationship between diabetes and depression are contradictory. The purpose of the present study was not to study causal relations of diabetes and depression but to examine the factors that may lead to depressive symptoms in already existing diabetes. But we also run additional longitudinal analyses that showed that the binary measure insulin dependence at T3 was significantly predicted by baseline anxiety/depression (*β* = |.38|; *p* < .05). We also found a cross-sectional direct significant association between insulin-dependence and anxiety/depression at T1 although there was no such relationship with the WHO-Five score (over and above all other measures in the model). These results suggest a relationship between physical and mental health that needs to be further examined. Also, previous studies have shown that one of the most important predictors of anxiety in persons with diabetes is the incidence or fear of hypoglycemia e.g. [28] which might affect the anxiety/depression subscale and explain why there is no such relationship with depressive symptoms in terms of the WHO-Five score in our data. The fear of hypoglycemia might be higher in persons who are insulin dependent as the use of insulin is associated with increased episodes of hypoglycemia [29]. Therefore the predictive value of the proposed model might increase and explain more variability in mental health when anxiety-specific measures like the Hypoglycaemia Fear Survey [30] are included.

In summary, the results of the current study show that subjective health constructs (FH, SRH) account for a considerable amount of the variance in depressive symptoms in individuals with diabetes. It appears that the perception of impairment and health affect emotional well-being and depressive symptoms more directly than physical correlated of diabetes. As a consequence, subjective health constructs should receive more attention from health practitioners in diabetes care. Individuals’ perceptions of their health status rather than objective indicators of PH are what may be most strongly related to depressive symptoms in diabetes.

### Comparison with existing literature

There is a substantial gap between research findings regarding the relationship of diabetes and depression and general practice. It is well-known that depressive symptoms are not recognized in general practice in about half the general patients and especially in patients with diabetes [31]. Even if recognized, most cases are not treated according to the guidelines for treatment of depressive disorders [32]. Previous studies recommend a monitoring of subclinical or minor depression for people with type 2 diabetes [33–35]. We propose to include preliminary assessments of SRH into regular diabetes screening, monitoring of the development, and, if indicated, address the issue with the patient or refer him or her to a psychologist for further depression screening. e.g. [8, 36].

There have been a few approaches to improve treatment of individuals with diabetes and depression. Behavioral approaches to improve patients’ glycemic control could show that a stress management program can result in benefits for patients with type 2 diabetes e.g. [37]. Also, for example, Osborn et al. successfully developed a training program for practitioners working with patients with depression and diabetes [38]. More and further approaches in education of patients and health practitioners are necessary. According to the current results, interventions that aim to reduce the perceived functional impairment could thereby be effective. This was also the conclusion of a recent meta-analysis that showed how strongly activity-restriction and depression were correlated in medical patients [39]. From a psychological perspective two possible approaches to address impairment known as structural and behavioral prevention [40] have been discussed. Structural prevention in this regard could include improving access to diabetes-specific health support such as medical foot care or improved food-labels to prevent adverse effects of inappropriate nutrition. The behavioral approach comprises elements of cognitive behavioral therapy such as cognitive reframing that might aim on emphasizing on aspects that are less impaired or reducing the perceived importance of specific functional impairments. In addition, training of appropriate disease management and self-care and thereby promoting diabetes literacy might be effective in reducing depressive symptoms in individuals with diabetes.

### Limitations

The results only apply to clinically diagnosed diabetes and therefore might not be valid for undiagnosed diabetes as we cannot assess how knowing about a certain diagnosis and probably be treated accordingly might affect health perception and behavior. Also, we were not able to control for the duration of diabetes, which might be an important factor of perceived physical impairment and SRH and should be included in future studies. The WHO-5 is a questionnaire that assesses general well-being and can be used as a screening test for clinical depression [23, 41], but it is not a standardized clinical interview. Therefore our data does not provide a reliable clinical classification of depressive symptoms. More measures of mental health should be included in future studies to clarify the mental health domain.

The number of comorbid diagnoses seems to be a promising variable to determine disease burden and overall physical health. Unfortunately we did not have enough information to calculate, for example, a Charlson Comorbidity Index, which would be even more meaningful in terms of physical health as it is a strong predictor of mortality.

Another limitation of our study is the relatively high drop-out over time in the longitudinal part of the study. We addressed the problem of missing data by using FIML estimation, which allowed us to include all available data points in the analysis. FIML is currently the state-of-the art in missing data analysis, as it allows retaining high statistical power in the presence of missing data [42]. Furthermore, by including auxiliary variables in the analyses (e.g., the Time-1 variables in the case of longitudinal analyses), bias is reduced relative to listwise deletion or other ad hoc missing data handling strategies. In the case of our longitudinal analyses, the covariance coverage remained relatively high (most covariance coverage values in terms of the proportion of data present to estimate a given variance or covariance were in the 60 to 70% range, with only a few values falling below 50% and no values falling below 40%, indicating that there was enough information available to estimate the path coefficients reliably in our longitudinal model. Nonetheless, it would be desirable to obtain more complete data in future studies if possible.

The strong associations between FH and SRH indicators and depressive symptoms may in part be explained by shared method effects, given that almost all these constructs were based on self-report measures. This may have led to an overestimation of the amount of explained variability in depressive symptoms due to shared method variance. Future studies should attempt to obtain additional objective measures of these constructs to control for potential effects of shared method variance. Furthermore, one could argue that the indicators of depressive symptoms and SRH share some conceptual similarity, and that this may explain the strong associations between SRH and depression in our study. On the other hand, Kudielka et al. [43] provided support for the argument that although depression and SRH are related concepts, they constitute distinct psychological entities. In our study, we found strong correlations between depression and measures of SRH, although all correlations were < .6, indicating a substantial amount of discriminant validity.

## Conclusions

The current results indicate that physical symptoms have only weak direct effects on depression, whereas the subjective ratings of health (SRH and FH) are strongly related to emotional well-being and depressive symptoms. Therefore, practitioners should be trained to pay more attention to the individual and potentially dysfunctional perception of the chronic disease. Our results contribute to the growing body of research that regards SRH as an important measure that might help identifying patients who require an early intervention.

### Endnote

^a^Individuals with available HbA_1c_ scores are significantly older (p < .05), report a significantly higher SRH (p < .001), HrQoL (p < .001) and emotional well-being (p < .001) and are more likely to be insulin dependent (p < .001) than individuals without available HbA_1c_ (48.7% vs. 33.4%).

## Electronic supplementary material

Additional file 1: **Longitudinal results.** (DOCX 97 KB)
