# Supplementary material for: Functional and self-rated health mediate the association between physical indicators of diabetes and depressive symptoms
Source: BMC Fam Pract. 2014 Sep 20;15:157. doi: 10.1186/1471-2296-15-157 (PMC4262980; doi:10.1186/1471-2296-15-157)
Supplement: Supplementary file 1 — Additional file 1: Longitudinal results. (DOCX 97 KB) [file 12875_2014_1131_MOESM1_ESM.docx]

Additional file 1: Table S1

*Longitudinal results*

|  | Paths | *B* | *SE*(*B*) | β | *p* | *R*^2^ | *SEE* |
| --- | --- | --- | --- | --- | --- | --- | --- |
| T2 | Mobility T2 on |  |  |  |  | .46 | .37 |
|  | Gender | .02 | .02 | -.03 | .287 |  |  |
|  | Age | .00 | .00 | -.10 | .001 |  |  |
|  | Number of diagnoses | .01 | .01 | -.10 | .037 |  |  |
|  | BMI | .01 | .00 | .81 | <.001 |  |  |
|  | Insulin-dependence | .07 | .02 | .00 | <.001 |  |  |
|  | Blood glucose level | .00 | .01 | -.02 | .312 |  |  |
|  |  |  |  |  |  |  |  |
|  | Mobility T1 | .45 | .02 | .02 | .214 |  |  |
|  | Self-Care T1 | .03 | .03 | .04 | .279 |  |  |
|  | Usual Activities T1 | .10 | .02 | -.01 | <.001 |  |  |
|  | Pain/Discomfort T1 | .08 | .02 | .04 | <.001 |  |  |
|  |  |  |  |  |  |  |  |
|  | SRH T1 | -.02 | .01 | .01 | .002 |  |  |
|  |  |  |  |  |  |  |  |
|  | Depressive Symptoms (WHO) T1 | -.00 | .00 | .03 | .111 |  |  |
|  | Depressive Symptoms (Anxiety/Depression) T1 | -.03 | .02 | .00 | .201 |  |  |
|  | Self-Care T2 on |  |  |  |  | .46 | .29 |
|  | Gender | -.01 | .02 | .02 | .661 |  |  |
|  | Age | .00 | .00 | .06 | .001 |  |  |
|  | Number of diagnoses | .00 | .01 | .04 | .835 |  |  |
|  | BMI | .00 | .00 | .07 | .862 |  |  |
|  | Insulin-dependence | .03 | .02 | .07 | .051 |  |  |
|  | Blood glucose level | -.02 | .01 | .00 | .028 |  |  |
|  |  |  |  |  |  |  |  |
|  | Mobility T1 | .02 | .02 | .45 | .271 |  |  |
|  | Self-Care T1 | .58 | .02 | .02 | <.001 |  |  |
|  | Usual Activities T1 | .08 | .02 | .11 | <.001 |  |  |
|  | Pain/Discomfort T1 | -.01 | .01 | -.02 | .328 |  |  |
|  |  |  |  |  |  |  |  |
|  | SRH T1 | -.02 | .01 | -.07 | .003 |  |  |
|  |  |  |  |  |  |  |  |
|  | Depressive Symptoms (WHO) T1 | -.00 | .00 | -.04 | .173 |  |  |
|  | Depressive Symptoms (Anxiety/Depression) T1 | .02 | .02 | .03 | .213 |  |  |
|  | Usual Activities T2 on |  |  |  |  | .51 | .40 |
|  | Gender | -.00 | .02 | -.00 | .968 |  |  |
|  | Age | -.00 | .00 | -.02 | .389 |  |  |
|  | Number of diagnoses | .02 | .01 | .05 | .004 |  |  |
|  | BMI | .00 | .00 | .01 | .500 |  |  |
|  | Insulin-dependence | .03 | .02 | .03 | .107 |  |  |
|  | Blood glucose level | .01 | .01 | .01 | .631 |  |  |
|  |  |  |  |  |  |  |  |
|  | Mobility T1 | .11 | .02 | .10 | <.001 |  |  |
|  | Self-Care T1 | .22 | .03 | .14 | <.001 |  |  |
|  | Usual Activities T1 | .39 | .02 | .38 | <.001 |  |  |
|  | Pain/Discomfort T1 | .07 | .02 | .08 | <.001 |  |  |
|  |  |  |  |  |  |  |  |
|  | SRH T1 | -.04 | .01 | -.13 | <.001 |  |  |
|  |  |  |  |  |  |  |  |
|  | Depressive Symptoms (WHO) T1 | -.00 | .00 | -.05 | .040 |  |  |
|  | Depressive Symptoms (Anxiety/Depression) T1 | .03 | .02 | .03 | .103 |  |  |
|  | Pain/Discomfort T2 on |  |  |  |  | .50 | .42 |
|  | Gender | -.06 | .03 | -.04 | .018 |  |  |
|  | Age | .00 | .00 | .03 | .074 |  |  |
|  | Number of diagnoses | .02 | .01 | .04 | .041 |  |  |
|  | BMI | .01 | .00 | .06 | <.001 |  |  |
|  | Insulin-dependence | .01 | .02 | .01 | .496 |  |  |
|  | Blood glucose level | -.00 | .01 | -.01 | .790 |  |  |
|  |  |  |  |  |  |  |  |
|  | Mobility T1 | .10 | .03 | .09 | <.001 |  |  |
|  | Self-Care T1 | -.06 | .03 | -.04 | .040 |  |  |
|  | Usual Activities T1 | .05 | .03 | .05 | .045 |  |  |
|  | Pain/Discomfort T1 | .56 | .02 | .56 | <.001 |  |  |
|  |  |  |  |  |  |  |  |
|  | SRH T1 | -.02 | .01 | -.06 | .008 |  |  |
|  |  |  |  |  |  |  |  |
|  | Depressive Symptoms (WHO) T1 | -.00 | .00 | -.06 | .010 |  |  |
|  | Depressive Symptoms (Anxiety/Depression) T1 | -.03 | .02 | -.03 | .182 |  |  |
|  | SRH T2 on |  |  |  |  | .48 | 1.40 |
|  | Gender | .05 | .09 | .01 | .597 |  |  |
|  | Age | -.01 | .01 | -.04 | .057 |  |  |
|  | Number of diagnoses | -.06 | .03 | -.05 | .012 |  |  |
|  | BMI | -.00 | .01 | -.00 | .827 |  |  |
|  | Insulin-dependence | -.17 | .07 | -.04 | .024 |  |  |
|  | Blood glucose level | -.05 | .05 | -.02 | .287 |  |  |
|  |  |  |  |  |  |  |  |
|  | Mobility T1 | -.21 | .09 | -.06 | .014 |  |  |
|  | Self-Care T1 | -.13 | .11 | -.03 | .229 |  |  |
|  | Usual Activities T1 | -.11 | .09 | -.03 | .192 |  |  |
|  | Pain/Discomfort T1 | -.34 | .07 | -.10 | <.001 |  |  |
|  |  |  |  |  |  |  |  |
|  | SRH T1 | .45 | .03 | .05 | <.001 |  |  |
|  |  |  |  |  |  |  |  |
|  | Depressive Symptoms (WHO) T1 | .01 | .00 | .15 | <.001 |  |  |
|  | Depressive Symptoms (Anxiety/Depression) T1 | -.01 | .01 | -.00 | .914 |  |  |
|  | Depressive Symptoms (WHO) T2 on |  |  |  |  | .57 | 15.94 |
|  | Gender | .85 | 1.01 | .01 | .396 |  |  |
|  | Age | .01 | .06 | .00 | .840 |  |  |
|  | Number of diagnoses | -.72 | .28 | -.05 | .011 |  |  |
|  | BMI | -.08 | .08 | -.02 | .279 |  |  |
|  | Insulin-dependence | -.75 | .84 | -.02 | .370 |  |  |
|  | Blood glucose level | .91 | .53 | .03 | .088 |  |  |
|  |  |  |  |  |  |  |  |
|  | Mobility T1 | -1.73 | .98 | -.04 | .079 |  |  |
|  | Self-Care T1 | -2.84 | 1.28 | -.04 | .027 |  |  |
|  | Usual Activities T1 | .75 | .99 | .02 | .444 |  |  |
|  | Pain/Discomfort T1 | .04 | .84 | .00 | .967 |  |  |
|  |  |  |  |  |  |  |  |
|  | SRH T1 | 1.01 | .29 | .08 | <.001 |  |  |
|  |  |  |  |  |  |  |  |
|  | Depressive Symptoms (WHO) T1 | .60 | .02 | .60 | <.001 |  |  |
|  | Depressive Symptoms (Anxiety/Depression) T1 | -4.72 | .87 | -.11 | <.001 |  |  |
|  | Depressive Symptoms (Anxiety/Depression) T2 on |  |  |  |  | .50 | .37 |
|  | Gender | .01 | .02 | -.01 | .748 |  |  |
|  | Age | -.00 | .00 | -.02 | .401 |  |  |
|  | Number of diagnoses | -.00 | .01 | -.00 | .820 |  |  |
|  | BMI | -.00 | .00 | -.01 | .718 |  |  |
|  | Insulin-dependence | .00 | .02 | .00 | .986 |  |  |
|  | Blood glucose level | -.00 | .01 | -.00 | .881 |  |  |
|  |  |  |  |  |  |  |  |
|  | Mobility T1 | .02 | .02 | .02 | .476 |  |  |
|  | Self-Care T1 | .01 | .03 | -.01 | .749 |  |  |
|  | Usual Activities T1 | -.02 | .02 | -.02 | .483 |  |  |
|  | Pain/Discomfort T1 | .00 | .02 | -.00 | .870 |  |  |
|  |  |  |  |  |  |  |  |
|  | SRH T1 | -.01 | .01 | -.05 | .050 |  |  |
|  |  |  |  |  |  |  |  |
|  | Depressive Symptoms (WHO) T1 | -.00 | .00 | -.16 | <.001 |  |  |
|  | Depressive Symptoms (Anxiety/Depression) T1 | .56 | .02 | .58 | <.001 |  |  |
| T3 | Mobility T3 on |  |  |  |  | .53 | .35 |
|  | BMI T2 | -.00 | .00 | -.01 | .705 |  |  |
|  | Mobility T2 | .32 | .03 | .32 | <.001 |  |  |
|  | Self-Care T2 | .07 | .03 | .05 | .039 |  |  |
|  | Usual Activities T2 | .05 | .02 | .05 | .046 |  |  |
|  | Pain/Discomfort T2 | .01 | .02 | .02 | .556 |  |  |
|  |  |  |  |  |  |  |  |
|  | SRH T2 | .00 | .01 | .00 | .888 |  |  |
|  |  |  |  |  |  |  |  |
|  | Depressive Symptoms (WHO) T2 | -.00 | .00 | -.08 | .009 |  |  |
|  | Depressive Symptoms (Anxiety/Depression) T2 | .03 | .03 | .03 | .218 |  |  |
|  |  |  |  |  |  |  |  |
|  | Gender | -.01 | .02 | -.01 | .567 |  |  |
|  | Age | .00 | .00 | .06 | .002 |  |  |
|  | Number of diagnoses | .01 | .01 | .04 | .029 |  |  |
|  | BMI | .00 | .00 | .02 | .532 |  |  |
|  | Insulin-dependence | .05 | .02 | .05 | .005 |  |  |
|  | Blood glucose level | -.00 | .01 | -.00 | .950 |  |  |
|  |  |  |  |  |  |  |  |
|  | Mobility T1 | .03 | .02 | .30 | <.001 |  |  |
|  | Self-Care T1 | -.01 | .03 | -.01 | .658 |  |  |
|  | Usual Activities T1 | .03 | .02 | .04 | .123 |  |  |
|  | Pain/Discomfort T1 | .04 | .02 | .05 | .057 |  |  |
|  |  |  |  |  |  |  |  |
|  | SRH T1 | -.01 | .01 | -.02 | .408 |  |  |
|  |  |  |  |  |  |  |  |
|  | Depressive Symptoms (WHO) T1 | .00 | .00 | .06 | .041 |  |  |
|  | Depressive Symptoms (Anxiety/Depression) T1 | -.01 | .02 | -.01 | .592 |  |  |
|  | Self-Care T3 on |  |  |  |  | .53 | .30 |
|  | BMI T2 | -.00 | .00 | -.01 | .720 |  |  |
|  | Mobility T2 | -.04 | .02 | -.05 | .046 |  |  |
|  | Self-Care T2 | .42 | .03 | .38 | <.001 |  |  |
|  | Usual Activities T2 | .08 | .02 | .11 | <.001 |  |  |
|  | Pain/Discomfort T2 | .01 | .02 | .01 | .764 |  |  |
|  |  |  |  |  |  |  |  |
|  | SRH T2 | -.01 | .01 | -.06 | .030 |  |  |
|  |  |  |  |  |  |  |  |
|  | Depressive Symptoms (WHO) T2 | -.00 | .00 | -.08 | .013 |  |  |
|  | Depressive Symptoms (Anxiety/Depression) T2 | .04 | .02 | .05 | .064 |  |  |
|  |  |  |  |  |  |  |  |
|  | Gender | .02 | .02 | .02 | .193 |  |  |
|  | Age | .00 | .00 | .06 | .001 |  |  |
|  | Number of diagnoses | -.00 | .01 | -.00 | .817 |  |  |
|  | BMI | .00 | .00 | .02 | .422 |  |  |
|  | Insulin-dependence | .02 | .02 | .03 | .138 |  |  |
|  | Blood glucose level | .01 | .01 | .01 | .540 |  |  |
|  |  |  |  |  |  |  |  |
|  | Mobility T1 | .02 | .02 | .02 | .341 |  |  |
|  | Self-Care T1 | .28 | .03 | .24 | <.001 |  |  |
|  | Usual Activities T1 | .06 | .02 | .07 | .003 |  |  |
|  | Pain/Discomfort T1 | .00 | .02 | .00 | .991 |  |  |
|  |  |  |  |  |  |  |  |
|  | SRH T1 | -.00 | .01 | -.02 | .518 |  |  |
|  |  |  |  |  |  |  |  |
|  | Depressive Symptoms (WHO) T1 | .00 | .00 | .10 | .001 |  |  |
|  | Depressive Symptoms (Anxiety/Depression) T1 | .01 | .02 | .02 | .529 |  |  |
|  | Usual Activities T3 on |  |  |  |  | .54 | .39 |
|  | BMI T2 | -.00 | .00 | -.03 | .383 |  |  |
|  | Mobility T2 | .07 | .03 | .06 | .010 |  |  |
|  | Self-Care T2 | .18 | .04 | .12 | <.001 |  |  |
|  | Usual Activities T2 | .30 | .03 | .30 | <.001 |  |  |
|  | Pain/Discomfort T2 | .03 | .03 | .03 | .230 |  |  |
|  |  |  |  |  |  |  |  |
|  | SRH T2 | -.01 | .01 | -.04 | .129 |  |  |
|  |  |  |  |  |  |  |  |
|  | Depressive Symptoms (WHO) T2 | -.00 | .00 | -.10 | .001 |  |  |
|  | Depressive Symptoms (Anxiety/Depression) T2 | -.01 | .03 | -.01 | .761 |  |  |
|  |  |  |  |  |  |  |  |
|  | Gender | -.03 | .02 | -.02 | .286 |  |  |
|  | Age | -.00 | .00 | -.01 | .619 |  |  |
|  | Number of diagnoses | .01 | .01 | .04 | .040 |  |  |
|  | BMI | .00 | .00 | .02 | .560 |  |  |
|  | Insulin-dependence | .02 | .02 | .02 | .320 |  |  |
|  | Blood glucose level | .02 | .01 | .04 | .059 |  |  |
|  |  |  |  |  |  |  |  |
|  | Mobility T1 | .08 | .03 | .07 | .002 |  |  |
|  | Self-Care T1 | .06 | .04 | .04 | .081 |  |  |
|  | Usual Activities T1 | .20 | .03 | .19 | <.001 |  |  |
|  | Pain/Discomfort T1 | .02 | .02 | .02 | .474 |  |  |
|  |  |  |  |  |  |  |  |
|  | SRH T1 | -.00 | .01 | -.01 | .822 |  |  |
|  |  |  |  |  |  |  |  |
|  | Depressive Symptoms (WHO) T1 | .00 | .00 | .05 | .097 |  |  |
|  | Depressive Symptoms (Anxiety/Depression) T1 | .03 | .03 | .02 | .333 |  |  |
|  | Pain/Discomfort T3 on |  |  |  |  | .51 | .42 |
|  | BMI T2 | -.00 | .00 | -.02 | .603 |  |  |
|  | Mobility T2 | .04 | .03 | .03 | .246 |  |  |
|  | Self-Care T2 | .05 | .04 | .03 | .208 |  |  |
|  | Usual Activities T2 | .05 | .03 | .05 | .079 |  |  |
|  | Pain/Discomfort T2 | .33 | .03 | .33 | <.001 |  |  |
|  |  |  |  |  |  |  |  |
|  | SRH T2 | -.02 | .01 | -.07 | .013 |  |  |
|  |  |  |  |  |  |  |  |
|  | Depressive Symptoms (WHO) T2 | .00 | .00 | -.01 | .678 |  |  |
|  | Depressive Symptoms (Anxiety/Depression) T2 | .02 | .03 | .02 | .475 |  |  |
|  |  |  |  |  |  |  |  |
|  | Gender | -.05 | .03 | -.03 | .049 |  |  |
|  | Age | .00 | .00 | .02 | .222 |  |  |
|  | Number of diagnoses | .01 | .00 | .03 | .181 |  |  |
|  | BMI | .01 | .00 | .04 | .175 |  |  |
|  | Insulin-dependence | .01 | .02 | .01 | .672 |  |  |
|  | Blood glucose level | -.01 | .01 | -.02 | .332 |  |  |
|  |  |  |  |  |  |  |  |
|  | Mobility T1 | .04 | .03 | .03 | .170 |  |  |
|  | Self-Care T1 | -.03 | .04 | -.02 | .404 |  |  |
|  | Usual Activities T1 | .01 | .03 | .01 | .758 |  |  |
|  | Pain/Discomfort T1 | .29 | .03 | .29 | <.001 |  |  |
|  |  |  |  |  |  |  |  |
|  | SRH T1 | .01 | .01 | .02 | .513 |  |  |
|  |  |  |  |  |  |  |  |
|  | Depressive Symptoms (WHO) T1 | -.00 | .00 | -.05 | .118 |  |  |
|  | Depressive Symptoms (Anxiety/Depression) T1 | -.03 | .03 | -.03 | .224 |  |  |
|  | SRH T3 on |  |  |  |  | .53 | 1.36 |
|  | BMI T2 | -.01 | .01 | -.04 | .261 |  |  |
|  | Mobility T2 | -.20 | .10 | -.05 | .049 |  |  |
|  | Self-Care T2 | -.09 | .13 | -.02 | .523 |  |  |
|  | Usual Activities T2 | .06 | .10 | .02 | .522 |  |  |
|  | Pain/Discomfort T2 | -.16 | .09 | -.05 | .083 |  |  |
|  |  |  |  |  |  |  |  |
|  | SRH T2 | .40 | .03 | .39 | <.001 |  |  |
|  |  |  |  |  |  |  |  |
|  | Depressive Symptoms (WHO) T2 | .01 | .00 | .08 | .016 |  |  |
|  | Depressive Symptoms (Anxiety/Depression) T2 | -.02 | .10 | -.01 | .844 |  |  |
|  |  |  |  |  |  |  |  |
|  | Gender | -.26 | .09 | -.05 | .003 |  |  |
|  | Age | .00 | .01 | -.01 | .776 |  |  |
|  | Number of diagnoses | -.06 | .03 | -.05 | .015 |  |  |
|  | BMI | .01 | .01 | .01 | .685 |  |  |
|  | Insulin-dependence | -.04 | .07 | -.01 | .604 |  |  |
|  | Blood glucose level | -.07 | .05 | -.03 | .133 |  |  |
|  |  |  |  |  |  |  |  |
|  | Mobility T1 | -.06 | .10 | -.02 | .525 |  |  |
|  | Self-Care T1 | .00 | .14 | .00 | .982 |  |  |
|  | Usual Activities T1 | -.08 | .09 | -.02 | .385 |  |  |
|  | Pain/Discomfort T1 | -.08 | .09 | -.02 | .374 |  |  |
|  |  |  |  |  |  |  |  |
|  | SRH T1 | .20 | .03 | .20 | <.001 |  |  |
|  |  |  |  |  |  |  |  |
|  | Depressive Symptoms (WHO) T1 | .00 | .00 | .04 | .225 |  |  |
|  | Depressive Symptoms (Anxiety/Depression) T1 | .03 | .10 | .01 | .768 |  |  |
|  | Depressive Symptoms (WHO) T3 on |  |  |  |  | .60 | 15.93 |
|  | BMI T2 | -.00 | .13 | -.00 | .977 |  |  |
|  | Mobility T2 | -2.49 | 1.21 | -.05 | .040 |  |  |
|  | Self-Care T2 | -.33 | 1.63 | -.01 | .838 |  |  |
|  | Usual Activities T2 | -1.33 | 1.16 | -.03 | .252 |  |  |
|  | Pain/Discomfort T2 | 1.30 | 1.07 | .03 | .222 |  |  |
|  |  |  |  |  |  |  |  |
|  | SRH T2 | .49 | .35 | .04 | .169 |  |  |
|  |  |  |  |  |  |  |  |
|  | Depressive Symptoms (WHO) T2 | .43 | .03 | .41 | <.001 |  |  |
|  | Depressive Symptoms (Anxiety/Depression) T2 | -2.14 | 1.21 | -.05 | .077 |  |  |
|  |  |  |  |  |  |  |  |
|  | Gender | .22 | 1.04 | .00 | .837 |  |  |
|  | Age | .03 | .06 | .01 | .579 |  |  |
|  | Number of diagnoses | -.61 | .30 | -.04 | .041 |  |  |
|  | BMI | .19 | .14 | .04 | .193 |  |  |
|  | Insulin-dependence | -.82 | .86 | -.02 | .340 |  |  |
|  | Blood glucose level | .22 | .55 | -.01 | .685 |  |  |
|  |  |  |  |  |  |  |  |
|  | Mobility T1 | -1.16 | 1.11 | -.02 | .299 |  |  |
|  | Self-Care T1 | -1.42 | 1.61 | -.02 | .378 |  |  |
|  | Usual Activities T1 | .74 | 1.09 | .02 | .497 |  |  |
|  | Pain/Discomfort T1 | -1.57 | 1.04 | -.02 | .133 |  |  |
|  |  |  |  |  |  |  |  |
|  | SRH T1 | .31 | .34 | .02 | .351 |  |  |
|  |  |  |  |  |  |  |  |
|  | Depressive Symptoms (WHO) T1 | .29 | .03 | .28 | <.001 |  |  |
|  | Depressive Symptoms (Anxiety/Depression) T1 | -.11 | 1.12 | -.00 | .923 |  |  |
|  | Depressive Symptoms (Anxiety/Depression) T3 on |  |  |  |  | .52 | .38 |
|  | BMI T2 | -.00 | .00 | -.01 | .261 |  |  |
|  | Mobility T2 | -.05 | .03 | -.05 | .049 |  |  |
|  | Self-Care T2 | .05 | .04 | .03 | .523 |  |  |
|  | Usual Activities T2 | .03 | .03 | .03 | .522 |  |  |
|  | Pain/Discomfort T2 | .01 | .02 | .01 | .083 |  |  |
|  |  |  |  |  |  |  |  |
|  | SRH T2 | -.00 | .01 | -.00 | <.001 |  |  |
|  |  |  |  |  |  |  |  |
|  | Depressive Symptoms (WHO) T2 | -.00 | .00 | -.15 | .016 |  |  |
|  | Depressive Symptoms (Anxiety/Depression) T2 | .39 | .03 | .38 | .844 |  |  |
|  |  |  |  |  |  |  |  |
|  | Gender | -.02 | .02 | -.02 | .003 |  |  |
|  | Age | -.00 | .00 | -.03 | .148 |  |  |
|  | Number of diagnoses | -.01 | .01 | -.02 | .305 |  |  |
|  | BMI | -.00 | .00 | -.03 | .301 |  |  |
|  | Insulin-dependence | -.01 | .02 | -.01 | .590 |  |  |
|  | Blood glucose level | .01 | .01 | .01 | .489 |  |  |
|  |  |  |  |  |  |  |  |
|  | Mobility T1 | .00 | .03 | .00 | .873 |  |  |
|  | Self-Care T1 | .01 | .04 | .01 | .689 |  |  |
|  | Usual Activities T1 | .02 | .02 | .02 | .425 |  |  |
|  | Pain/Discomfort T1 | .03 | .02 | .03 | .180 |  |  |
|  |  |  |  |  |  |  |  |
|  | SRH T1 | .01 | .01 | .04 | .162 |  |  |
|  |  |  |  |  |  |  |  |
|  | Depressive Symptoms (WHO) T1 | .00 | .00 | -.02 | .470 |  |  |
|  | Depressive Symptoms (Anxiety/Depression) T1 | .26 | .03 | -.03 | <.001 |  |  |

*Note*. *B* = unstandardized path coefficient. *SE* = standard error; β = standardized path coefficient; *SEE* = standard error of estimate (estimated standard deviation of the residual variable).
